# A Case of Parinaud Oculoglandular Syndrome in Which *Bartonella* DNA Was Detected in the Cornea and Conjunctiva by Polymerase Chain Reaction

**DOI:** 10.3390/medicina60091425

**Published:** 2024-08-31

**Authors:** Junya Saito, Akira Machida, Daisuke Inoue, Masumi Suzuki Shimizu, Kohsuke Matsui, Kohei Harada, Mao Kusano, Yasser Helmy Mohamed, Masafumi Uematsu

**Affiliations:** 1 Department of Ophthalmology and Visual Sciences, Graduate School of Biomedical Sciences, Nagasaki University, 1-7-1 Sakamoto, Nagasaki 852-8501, Nagasaki, Japan; junya84237@gmail.com (J.S.);; 2 Department of Infectious Diseases, Nagasaki University Hospital, 1-7-1 Sakamoto, Nagasaki 852-8102, Nagasaki, Japan

**Keywords:** cat-scratch disease, Parinaud oculoglandular syndrome, *Bartonella henselae*

## Abstract

*Background and Objectives*: Parinaud oculoglandular syndrome (POS) is unilateral granulomatous follicular conjunctivitis with ipsilateral afferent lymphadenopathy, primarily caused by cat-scratch disease, tularemia, and sporotrichosis. We report a case of POS in which *Bartonella* DNA was detected using polymerase chain reaction (PCR) in corneal and conjunctival specimens. *Methods*: A 29-year-old man, who started keeping a stray cat two months prior, became aware of right preauricular lymphadenopathy and right ocular conjunctival hyperemia one month prior. Subsequently, he developed a fever of approximately 37.9 °C, with a purulent ocular discharge appearing 1 week before being referred to our department for a detailed ophthalmological examination. The patient’s right eye showed hyperemia and edema in the bulbar conjunctiva, along with palpebral conjunctival hyperemia, follicles, and white ulcers. Two weeks later, his serum IgM titer for *Bartonella henselae* was 1:20, and *Bartonella* DNA was detected by PCR in the corneal and conjunctival specimens. Based on these findings, the patient was diagnosed with POS caused by cat-scratch disease (CSD). Oral doxycycline, rifampicin, topical gatifloxacin, betamethasone phosphate, and erythromycin eye ointments were prescribed. *Results*: After 2 weeks of oral treatment and 2 months of eye drop treatment, the deterioration of the cornea and conjunctiva improved when the patient recovered good visual acuity. *Conclusions*: PCR assays of corneal and conjunctival specimens are useful for the diagnosis of CSD presenting with POS. These results suggested that *Bartonella* may be directly involved in the ocular surface pathogenesis of POS.

## 1. Introduction

Parinaud oculoglandular syndrome (POS), first reported by Parinaud in 1889, manifests as acute unilateral granulomatous conjunctivitis with ipsilateral lymphadenitis [1,2]. POS is reportedly caused by cat-scratch disease (CSD), tularemia, sporotrichosis, syphilis, and other diseases. POS is mainly diagnosed based on serum antibody titers and lymph node biopsies [3]. The causative diseases of POS are diverse, and a misdiagnosis may result in a worsening of the condition. Therefore, a reliable diagnosis is of great importance, as it is crucial for the appropriate treatment and management of the condition. Our case report describes a patient with POS caused by CSD in whom *Bartonella* DNA was detected by polymerase chain reaction (PCR) in corneal and conjunctival specimens.

## 2. Case Report

A 29-year-old man without a history of ocular disease visited our outpatient department with conjunctivitis in the right eye and a fever that had been present for approximately a week. He became aware of right preauricular lymphadenopathy after he started keeping a stray cat two months prior, with right conjunctival hyperemia observed one month prior.

**Figure 1:**
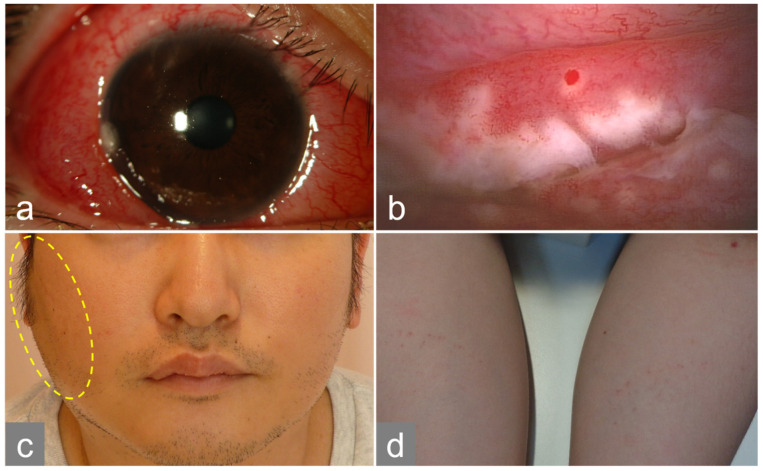
Photograph taken during the initial visit to our department. Hyperemia and edema were observed in the right ocular conjunctiva and dellen on the temporal side of the cornea (**a**). White lesions with ulceration, follicles, and hemorrhagic plaques were observed in the right lower eyelid conjunctiva (**b**). Right buccal lymphadenopathy (circled in yellow) and scratches on both forearms were also observed (**c**,**d**).

**Figure 2:**
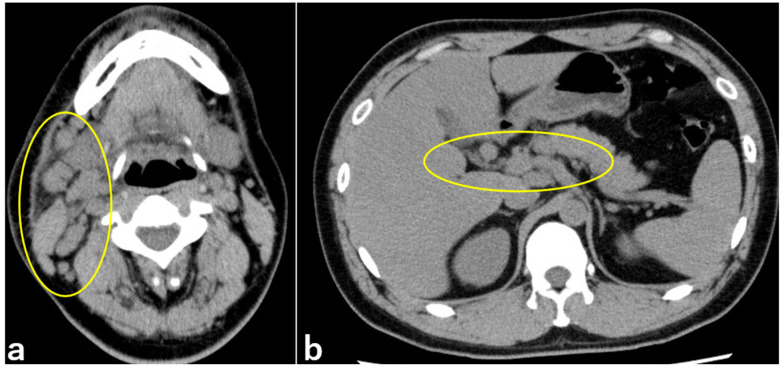
Computed tomography taken during the initial visit to our department. Lymphadenopathy (circled in yellow) was observed in the right parotid gland (**a**) and hilar region (**b**).

At the initial visit, the best-corrected visual acuity was 20/25 in the right eye (OD) and 20/20 in the left eye (OS). Intraocular pressure was 14.7 mmHg OD and 13.7 mmHg OS. Slit-lamp examination revealed unilateral bulbar conjunctival hyperemia, palpebral conjunctival follicles, and white ulcers in the right eye. Corneal dellen was noted on the temporal side of the right cornea (Figure 1a,b). Few anterior chamber cells were observed in the right eye. The lens and ocular fundus were normal in both eyes. Optical coherence tomography of the macula revealed normal findings. The patient’s temperature was 37.9 °C, his heart rate was 104 beats/min, and his blood pressure was 110/74 mmHg. Upon physical examination, cat scratches were observed on both forearms with preauricular and cervical lymphadenopathy on the right side (Figure 1c,d). Laboratory results revealed increased levels of white blood cells (9.4 × 10^3^ /µL), sedimentation rate (26 mm/hr), C-reactive protein (CRP) (3.89 mg/dL), alanine aminotransferase (58 U/L), alkaline phosphatase (121 U/L), and gamma-glutamyl transferase (113 U/L). Computed tomography revealed numerous enlarged lymph nodes extending from the right parotid gland to the supraclavicular fossa and swollen hilar lymph nodes (Figure 2a,b).

**Figure 3:**
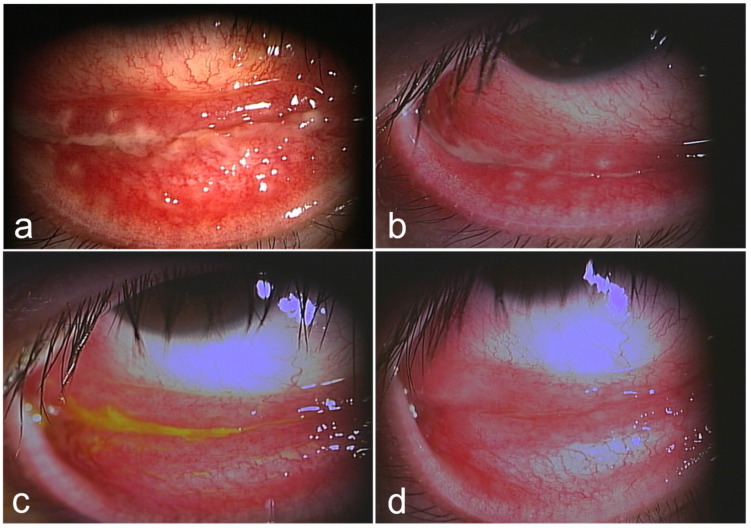
Lower eyelid conjunctival photographs over the course of treatment. The granuloma lesion on the right lower eyelid gradually shrank and improved ((**a**) 2 days; (**b**) 3 days; (**c**) 10 days; (**d**) 17 days).

Follicular conjunctivitis in the right eye and ipsilateral lymphadenitis led to a clinical diagnosis of POS. Owing to the 2-month history of cat ownership, CSD was suspected as the cause. Smear culture examination and PCR of the corneal epithelium around the corneal dellen and conjunctival white lesions were performed to identify the causative organism. In addition, serum antibody titers were measured for *Bartonella henselae*. Treatment with oral antibacterial medication (doxycycline 100 mg, rifampicin 600 mg daily), topical gatifloxacin, corticosteroids (fluorometholone 0.1%), and ocular ointment (erythromycin lactobionate) four times a day in the right eye was started. The patient’s general condition promptly improved, and oral treatment was completed within 2 weeks. CRP improved to 0.56 mg/dL 1 week after the start of treatment. The cervical lymphadenopathy disappeared 4 weeks after treatment initiation. Right eye conjunctivitis and corneal dellen gradually improved and disappeared without scarring at the time of the subsequent visit, 2 months after the start of the treatment (Figure 3). Ophthalmic treatment was continued for 2 months and then tapered off. Although both the smear and culture test results from the corneal and conjunctival specimens were negative, polymerase chain reaction (PCR) testing detected *Bartonella* DNA.

The quantitative polymerase chain reaction (qPCR) analysis was conducted at the Kobe City Eye Hospital [4,5]. Following the extraction of DNA from the samples, a qPCR reaction was conducted using a LightCycler 480 II (Roche Diagnostics, Basel, Switzerland) in a 96-well plate (20 µL/well). The reaction was initiated at 95 °C for 10 s, followed by 45 cycles at 95 °C for 5 s and 60 °C for 60 s. The calibration curves were generated with positive control DNA dilutions (10^6^, 10^4^, and 10^2^ copies/mL). Quantitative PCR testing revealed the presence of 2.40 × 10^1^ copies/µg of *Bartonella* in the corneal epithelium and 1.82 × 10^3^ copies/µg of *Bartonella* in the conjunctival white lesion samples.

The antibody titers of *Bartonella henselae* in serum were at a level of less than 20-fold for IgM and 64-fold for IgG at the first visit. At the subsequent visit, which took place 2 weeks later, the values were at a level of 20-fold for IgM and 128-fold for IgG. Based on the PCR test results, a definite diagnosis of POS due to CSD was made.

## 3. Discussion

There have been very few reports of *Bartonella henselae* detected from sources other than serum and lymph node biopsies in POS. In the field of ophthalmology, there has been only one report of a case in which *Bartonella henselae* DNA was detected by PCR on a swab of a conjunctival lesion [6]. Thus, our current patient is the second reported case in which *Bartonella* DNA was detected in conjunctival biopsy tissue. Furthermore, *Bartonella* DNA was detected in the corneal samples taken from the corneal dellen in this case. These findings suggest that PCR analysis of corneal and conjunctival specimens may be useful for confirming the diagnosis of POS due to CSD. Our results also suggest that *Bartonella henselae* may directly invade the conjunctiva and cornea, besides using the hematogenous and lymphatic routes due to scratching in the POS.

A definitive diagnosis of CSD is usually based on the isolation of *Bartonella henselae* from clinical specimens such as lymph nodes. However, clinical isolation and culture are often difficult to perform in routine practice. In recent years, CSD has often been diagnosed by measuring serum antibody titers of *Bartonella henselae*. Serologic diagnosis can be confirmed using a single serum sample if the IgM of *Bartonella henselae* is at a level of 20-fold and IgG is 256-fold. Even if a single serum sample did not meet these diagnostic criteria, a 4-fold or greater increase in IgG in paired sera was considered positive. In the present case, the IgM antibody level was 20-fold, so a single serum sample was used to confirm the diagnosis of CSD [7].

POS is a clinical form of unilateral granulomatous conjunctivitis with ipsilateral lymphadenitis originally described by Parinaud in 1889 [1,2]. Cat-scratch disease, tularemia, and sporotrichosis are the main causes [3]. CSD is a zoonosis transmitted from cats to humans by *Bartonella henselae* via cat flea. The scratched area subsequently develops papules and dark rashes, painful swelling of the lymph nodes, and common cold-like symptoms such as fever and malaise, which often spontaneously resolve [8]. Ocular complications found in CSD include anterior ocular lesions, as observed in this case; posterior ocular lesions of optic neuroretinitis; and focal retinochoroiditis. POS is seen in approximately 5% of patients with CSD and can cause lesions on either the ocular or eyelid conjunctiva, often resulting in necrosis or ulceration. It is also characterized by the swelling of the ipsilateral regional lymph nodes (e.g., preauricular, submandibular, cervical) [9].

CSD has a good prognosis that resolves spontaneously even without treatment. It is an intracellular parasite, and the administration of tetracyclines, macrolides, and new quinolones is considered to be effective [10,11]. Furthermore, while POS treatment has yet to be definitively established, eye drops or the oral administration of these antimicrobial agents may be effective in some cases. In more severe cases, oral rifampicin may be used in combination [12]. In our case, the symptoms were quickly relieved by oral antimicrobials (doxycycline 100 mg and rifampicin 600 mg daily), gatifloxacin, and steroid eye drops.

## 4. Conclusions

The current study examined a case of POS in which *Bartonella henselae* was both serologically detected and detected by the PCR of corneal and conjunctival specimens, which led to a definite diagnosis of CSD. These results suggest that performing PCR on corneal and conjunctival specimens may be useful in confirming the diagnosis of POS due to CSD and that *Bartonella henselae* may be directly involved in the ocular surface in the pathogenesis of POS.

## Data Availability

The datasets used during the current study are available from the corresponding authors upon reasonable request.
